# The -7351C/T Polymorphism in the TPA Gene and Ischemic Stroke Risk: A Meta-Analysis

**DOI:** 10.1371/journal.pone.0053558

**Published:** 2013-01-09

**Authors:** Xunsha Sun, Rong Lai, Jiaoxing Li, Man Luo, Yufang Wang, Wenli Sheng

**Affiliations:** Department of Neurology, First Affiliated Hospital, Sun Yat-sen University, Guangzhou, People’s Republic of China; University Medical Center Utrecht, The Netherlands

## Abstract

**Background:**

A number of studies assessed the association of tissue plasminogen activator(*TPA*) gene polymorphisms with ischemic stroke, but the results were contradictory. We aimed to explore the role of *TPA* -7351C/T SNP in the susceptibility to ischemic stroke through a meta-analysis.

**Methods:**

The PubMed, MEDLINE, EMBASE, China Biological Medicine Database and WANFANG DATA databases were searched until August 2012. The strict selection criteria and exclusion criteria were determined, and odds ratios (ORs) with 95% confidence intervals (CIs) were used to assess the strength of associations. Stroke subtype was determined using Trial of Org 10172 in Acute Treatment criteria (TOAST). Statistical analyses were performed using the STATA12.0 software.

**Results:**

A total of 2,299 ischemic stroke cases and 1,948 controls in seven case-control studies were included in the meta-analysis. Significant association between -7351C/T polymorphism in the TPA gene and ischemic stroke was observed in all comparison models (TT+CT versus CC, TT versus CT+CC and T versus C). In the subgroup analysis by ethnicity, TT homozygote carriers had a 142% increased risk of ischemic stroke compared with the C allele carriers among East-Asians (TT versus CT+CC: OR = 2.42, 95% CI = 1.07–5.48), but not in South-Asians and Caucasians, and significantly increased risks were found for T versus C among both East-Asians (OR = 1.33, 95% CI = 1.05–1.68) and Caucasians(OR = 1.16, 95% CI = 1.02–1.31). Further stratification for stroke subtype in three Caucasian studies showed the association between -7351C/T polymorphism and Large-artery atherosclerosis (LAA), but not Small-vessel occlusion (SVO) and Cardioembolism (CE).

**Conclusions:**

This meta-analysis suggested that the -7351C/T polymorphism in *TPA* gene would be a risk factor for ischemic stroke, especially among East-Asians compared with Caucasians, but not in South-Asians, and it may play a role in the pathogenesis of LAA in Caucasians, but not in SVO and CE.

## Introduction

The coagulation system and fibrinolysis system maintain a dynamic equilibrium under normal conditions. When pathologic processes overwhelm the regulatory mechanisms, excessive quantities of thrombin form, initiating thrombosis [1]. Thrombosis is a critical event in the arterial diseases associated with ischemic stroke [2], so any factors that influence the critical balance within the fibrinolysis system may be of particular relevance to the risk of ischemic stroke.

Endothelium-derived tissue plasminogen activator (TPA) is the primary mediator of local intravascular fibrinolysis [3]. Plasma TPA levels are regulated by an acute release of local TPA that is effected by genetic factors. A single nucleotide polymorphism, -7351C/T, in the enhancer region of the *TPA* gene was first identified in 2000 and shown to be associated with the TPA release rates [4]. It has subsequently been demonstrated to be correlated with myocardial infarction [5]. However, various studies which were designed to investigate the association between -7351C/T in *TPA* and ischemic stroke showed contradictory results. Jannes et al. found the TT genotype was significantly associated with ischemic stroke in Australian population (OR = 1.90; 95% CI = 1.01–3.60). Stratification for stroke subtype showed the association between the polymorphism and lacunar infarction(OR = 2.70; 95% CI = 1.10–6.70), but not other subtypes [6], yet other studies failed to replicate it [7], [8], [9], [10], [11], [12]. Many of these studies used relatively small sample sizes, and such studies may have missed true associations of modest effect, so a meta-analysis of these results is needed to explore the inconsistencies.

## Materials and Methods

### Identification of Studies

The PubMed, MEDLINE, EMBASE, China Biological Medicine Database and WANFANG DATA databases were searched for publications until August 2012 using the following terms: ‘ischemic stroke’ OR ‘cerebral infarction’ OR ‘cerebrovascular disease’ OR ‘stroke’ in combination with ‘tissue plasminogen activator’ or ‘*TPA*’ and ‘polymorphism’ OR ‘variant’. The search was performed without any restriction on language. Two investigators (X Sun and R Lai) independently reviewed study eligibility and reached a consensus on which studies to include for review. We also checked the reference of selected articles for any further relevant studies [13].

The selected studies had to be in accordance with the following major criteria: a) well-designed case-control studies to evaluate the association between *TPA* -7351C/T and ischemic stroke, b) ischemic stroke was confirmed by neuroimaging data (magnetic resonance imaging or computer tomography), c) containing useful genotype frequencies, d) the genotype distribution of controls in Hardy-Weinberg equilibrium (HWE).

Studies were excluded if one of the following existed: a) patients younger than 18 years old, b) the genotype frequencies or number not reported, c) animal studies, reviews, case reports, abstracts and family-based studies. For duplicate or overlapped publications, we choose the largest sample size or the most recent one.

### Data Extraction

The following information was extracted from the selected studies by two independent investigators, any discrepancy was resolved by discussion or a third author (Li): first authors, publication year, country in which the study was conducted, characteristics of cases and controls (mean age, distribution of gender and sample ethnicity), number of genotypes and total number of cases and controls.

### Statistical Analysis

The strength of the association between *TPA* -7351C/T gene polymorphism and risk of ischemic stroke was assessed by pooled odds ratios (ORs) and corresponding 95% confidence intervals (CIs). The pooled ORs were performed for dominant model (TT+CT versus CC), recessive model (TT versus CT+CC), and additive model (T versus C) respectively. Stratified analyses were also performed by ethnicity and subtypes of ischemic stroke according to the Trial of Org 10172 in Acute Stroke Treatment (TOAST) classification. The presence of heterogeneity was calculated by the Chi-square-based Q-test. If the *P*>0.1 of the Q-test which indicated heterogeneity was not observed among the studies, the fixed-effects model (Mantel-Haenszel method) was used to calculate the pooled ORs. Otherwise, the random-effects model was adopted. The Z test was used to determine the pooled OR with the significance set at *P*<0.05. Publication bias was tested by Begg’s funnel plot [14] and Egger’s test [15], the significance was set at the *P*<0.05 level. Sensitivity analysis was performed by sequentially excluding individual study to assess the stability of the results. HWE was tested by Pearson’s X^2^ test (*P*<0.05 means deviated from HWE). STATA 12.0 software was used to perform all statistical analyses (StataCorp, College Station, TX, USA).

## Results

### Eligible Studies

**Figure 1:**
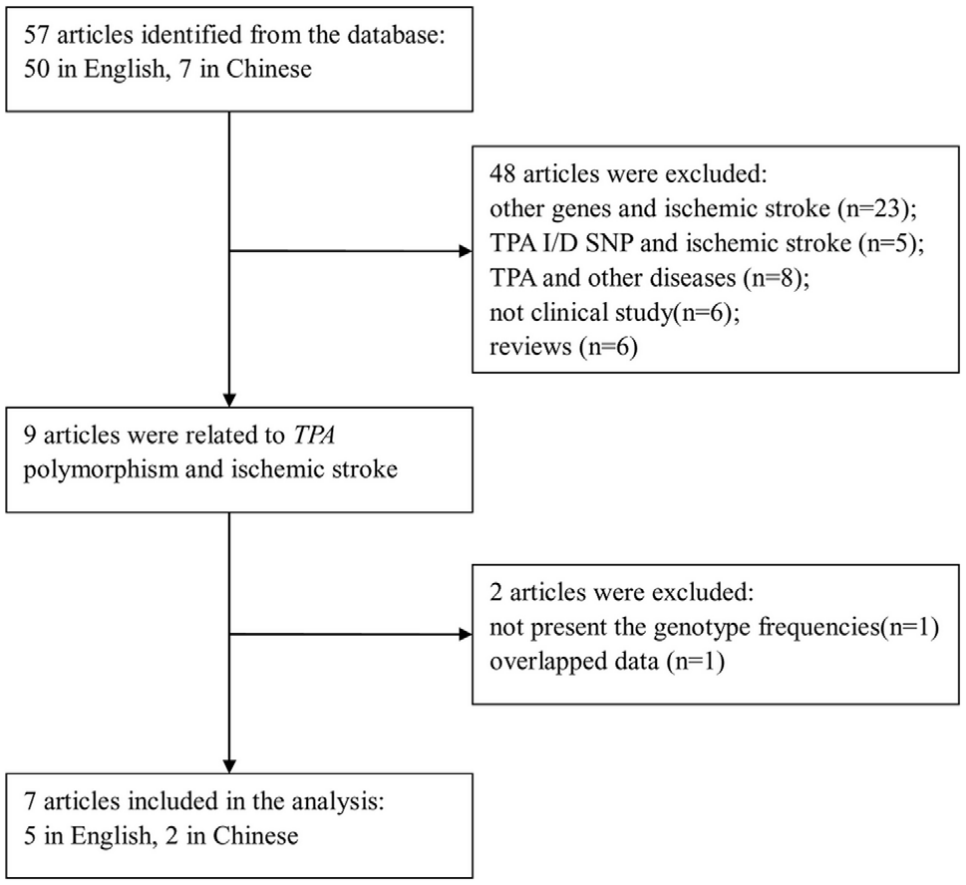
Flow of study identification, inclusion, and exclusion.

A total of fifty-seven papers were obtained by the literature search, among which seven fit the inclusion criteria [6], [7], [8], [11], [12], [16], [17], including 2,299 ischemic stroke cases and 1,948 controls (Figure 1). The data collected from the related studies was summarized in Table 1. Among the seven studies associated with -7351C/T SNP in *TPA* and ischemic stroke, two were conducted in East-Asians, one in South-Asians and four in Caucasians. In addition, three of four Caucasian studies reported ischemic stroke subtypes information according to the TOAST classification [18], other studies were classified by the Oxfordshire Community Stroke Project (OCSP) classification system [19]or without further subtypes analysis. Compared with 337 cases and 470 controls using the OCSP classification, there were 1306 cases and 832 controls involved in the TOAST classification system in our meta-analysis. So the stratified analyses were performed by subtypes of ischemic stroke according to the TOAST, including three major etiological types commonly distinguished: Large-artery atherosclerosis(LAA), Small-vessel occlusion (SVO) and Cardioembolism(CE). The distributions of genotype in these subtypes were shown in Table 2.

**Table 1:** Characteristics of the studies included in the meta-analysis.

Study	Country	Ethnicity	Mean age ofIS/control, years	Males ofIS/control,%	IS			Control			Sample size (IS/control)	Subtype	HWE
					CC	CT	TT	CC	CT	TT			
Jannes(2004)	Australia	Caucasian	73.4±11.6/73.6±12.0	55/56	74	83	23	137	136	28	180/301	OCSP	0.487
Jood(2005)	Sweden	Caucasian	56.0±10.0/56.0±10.0	64/64	259	277	64	278	266	56	600/600	TOAST	0.504
Wang(2006)	China	East-Asian	70.2±12/70.0±11.6	41/69	61	37	42	59	60	14	140/133	NG	0.827
Geng(2008)	China	East-Asian	70.6±13.2/70.8±13.1	59/60	66	70	21	75	79	15	157/169	OCSP	0.365
Maguire(2011)	Australia	Caucasian	74.9±13.2/65.9±7.5	54/47	273	271	66	84	86	14	610/184	TOAST	0.785
Babu(2012)	India	South-Asian	49.3±17.3/40.0±16.8	70/70	238	227	51	259	216	38	516/513	NG	0.441
Tuttolomondo(2012)	Italy	Caucasian	71.9±9.75/71.4±7.45	45/16	17	63	16	21	21	6	96/48	TOAST	0.834

IS: ischemic stroke. HWE: Hardy-Weinberg equilibrium of control. NG: not given.

OCSP: Oxfordshire Community Stroke Project. TOAST: Trial of Org 10172 in Acute Treatment.

**Table 2:** Distributions of TPA genotype among patients with ischemic stroke subtype and controls.

Subtype	Sample size (IS/control)	IS			Control		
		CC	CT	TT	CC	CT	TT
LAA							
Jood(2005)	73/600	27	37	9	278	266	56
Maguire(2011)	144/184	56	70	18	84	86	14
Tuttolomondo(2012)	37/48	2	27	8	21	21	6
SVO							
Jood(2005)	124/600	55	60	9	278	266	56
Maguire(2011)	120/184	55	52	13	84	86	14
Tuttolomondo(2012)	34/48	10	19	5	21	21	6
CE							
Jood(2005)	98/600	47	40	11	278	266	56
Maguire(2011)	179/184	87	76	16	84	86	14
Tuttolomondo(2012)	25/48	5	17	3	21	21	6

IS: ischemic stroke.

### Meta-analysis Results

**Figure 2:**
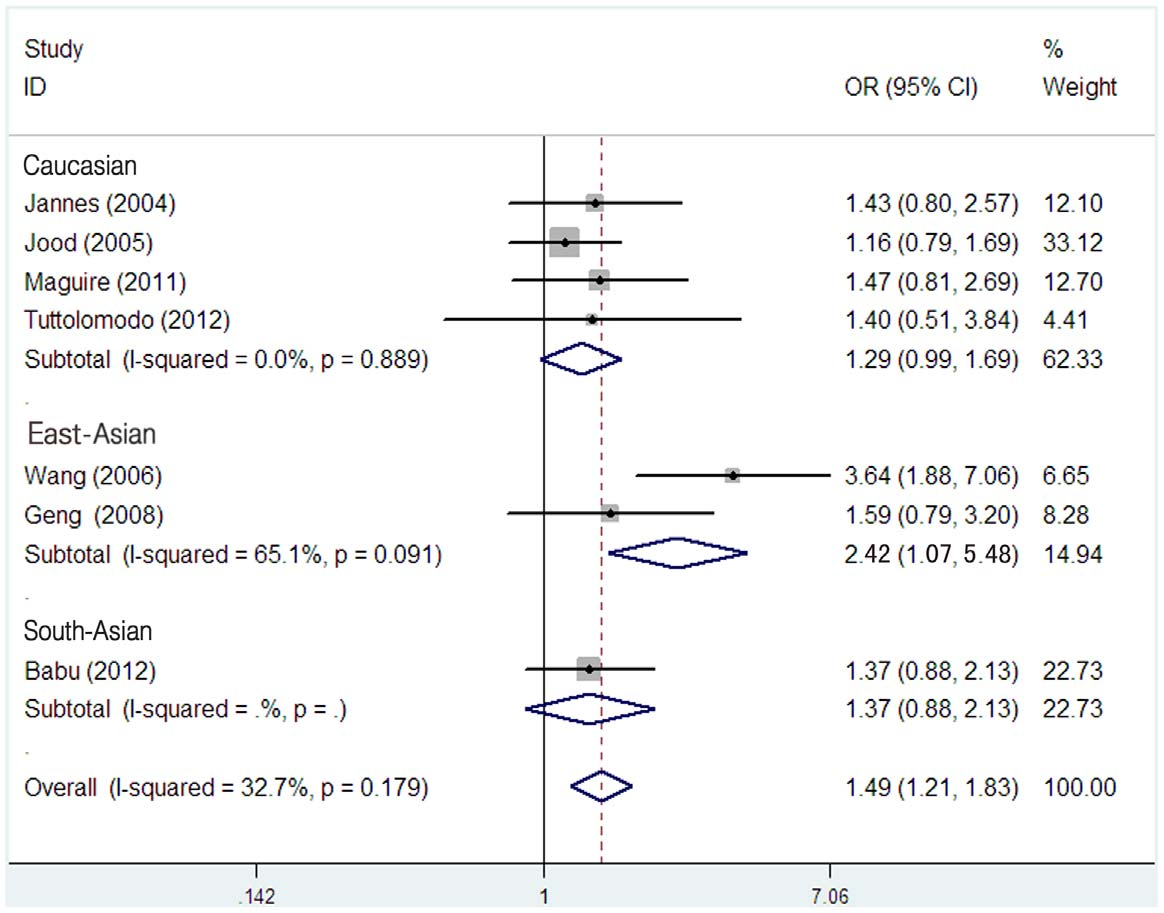
Meta-analysis for the association between the *TPA* -7351C/T polymorphism and ischemic stroke (TT vs CT+CC) among different ethnicity populations.

**Figure 3:**
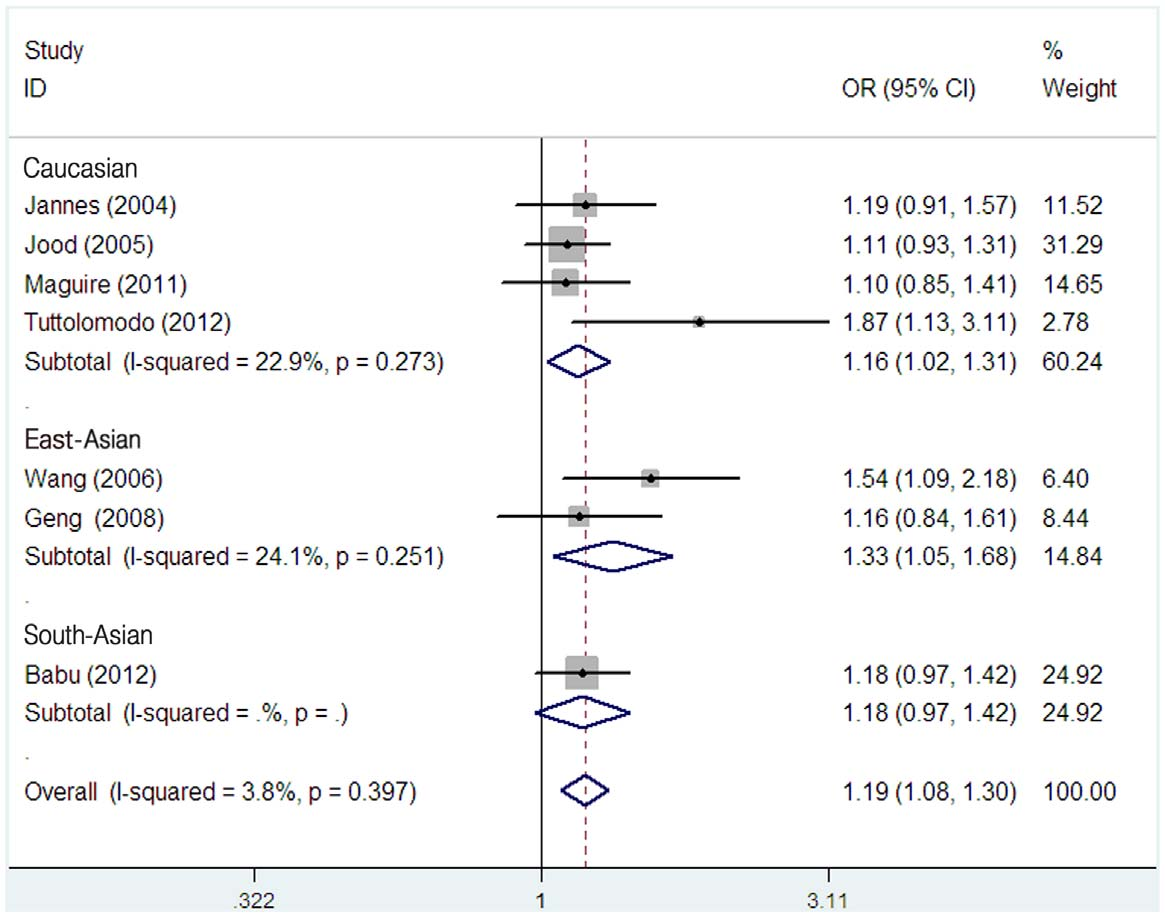
Meta-analysis with fixed effects model for the association between the *TPA* -7351C/T polymorphism and ischemic stroke (T vs C) among different ethnicity populations.

As shown in Table 3, *TPA* -7351C/T polymorphism was significantly associated with ischemic stroke in all comparison models (TT+CT versus CC, OR = 1.17, 95% CI = 1.03–1.32; TT versus CT+CC, OR = 1.49, 95% CI = 1.21–1.83; T versus C, OR = 1.19, 95% CI = 1.08–1.30). However, in the stratified analysis by ethnicity, significantly increased risk of ischemic stroke was found among East-Asians in recessive model (TT versus CT+CC, OR = 2.42, 95% CI = 1.07–5.48) (Figure 2) and additive model (T versus C, OR = 1.33, 95% CI = 1.05–1.68)(Figure 3), but not in dominant model (TT+CT versus CC, OR = 1.07, 95% CI = 0.77–1.48). In contrast, no significant results were observed in South-Asians (TT+CT versus CC, OR = 1.19, 95% CI = 0.93–1.52; TT versus CT+CC, OR = 1.37, 95% CI = 0.88–2.13; T versus C, OR = 1.18, 95% CI = 0.97–1.42) and Caucasians (TT+CT versus CC, OR = 1.28, 95% CI = 0.94–1.76; TT versus CT+CC, OR = 1.29, 95% CI = 0.99–1.69), except for the additive effect in Caucasians (T versus C, OR = 1.16, 95% CI = 1.02–1.31).

**Table 3:** Summary of results from different comparative genetic models.

Variables	n^a^	Case/control	TT+CT VS CC		TT VS CT+CC		T VS C	
			OR (95% CI)	P^b^	OR (95% CI)	P^b^	OR (95% CI)	P^b^
Total	7	2299/1948	1.17(1.03–1.32)	0.017	1.49(1.21–1.83)	0.0001	1.19(1.08–1.30)	0.0004
Ethnicities								
Caucasian	4	1486/1133	1.28(0.94–1.76)^c^	0.122	1.29(0.99–1.69)	0.062	1.16(1.02–1.31)	0.019
East-Asian	2	297/302	1.07(0.77–1.48)	0.687	2.42(1.07–5.48)^c^	0.033	1.33(1.05–1.68)	0.019
South-Asian	1	516/513	1.19(0.93–1.52)	0.162	1.37(0.88–2.13)	0.159	1.18(0.97–1.42)	0.091
Subtype								
LAA	3	254/832	2.05(0.94–4.50)^c^	0.073	1.61(1.00–2.59)	0.048	1.43(1.15–1.79)	0.002
SVO	3	278/832	1.10(0.83–1.47)	0.494	1.04(0.64–1.69)	0.872	1.07(0.86–1.32)	0.557
CE	3	96/832	1.00(0.75–1.33)	0.983	1.18(0.73–1.91)	0.497	1.03(0.83–1.28)	0.777

a Number of case-control studies.

b P value for z-test.

c Random-effects mode.

**Figure 4:**
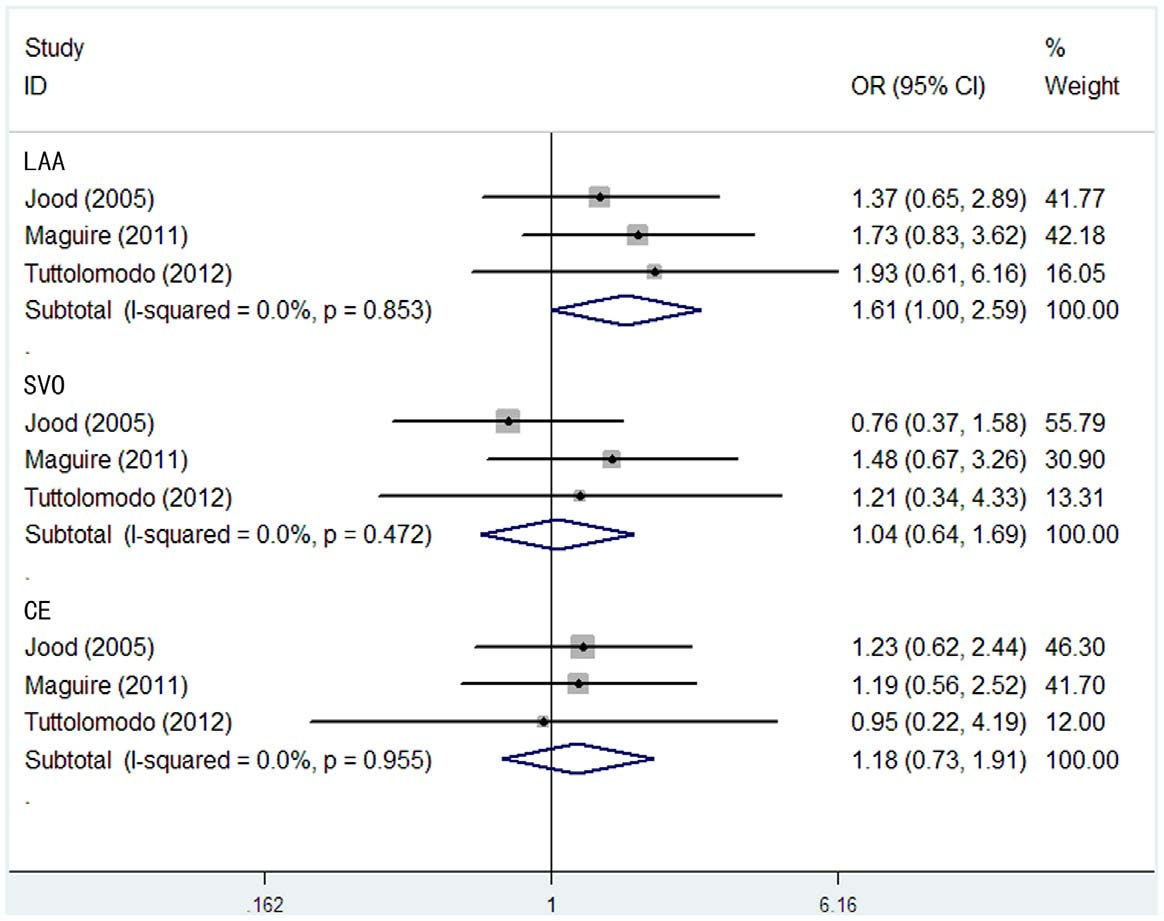
Meta-analysis with fixed-effects model for the association between the *TPA* -7351C/T polymorphism and ischemic stroke (TT vs CT+CC) among different subtypes of ischemic stroke according to the TOAST classification.

**Figure 5:**
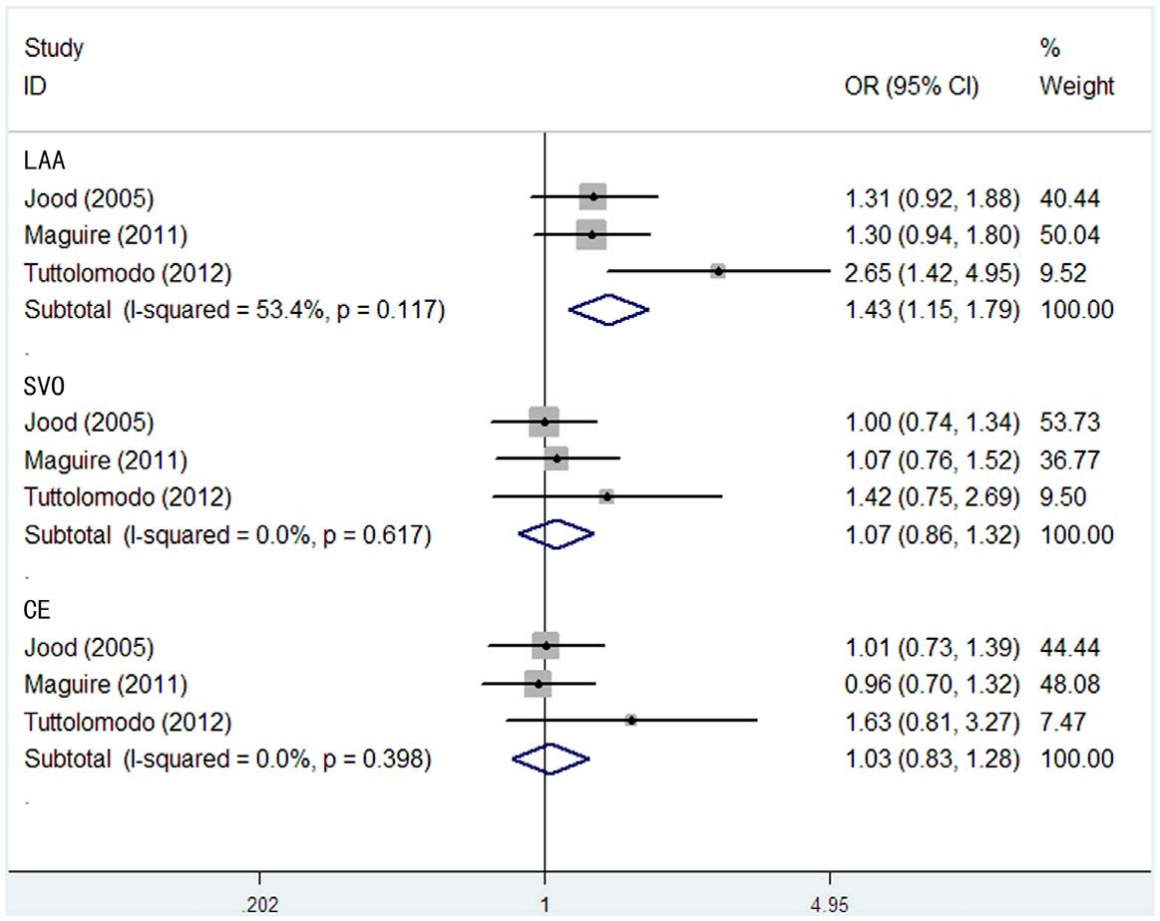
Meta-analysis with fixed-effects model for the association between the *TPA* -7351C/T polymorphism and ischemic stroke (T vs C) among different subtypes of ischemic stroke according to the TOAST classification.

In the stratified analysis by subtypes of ischemic stroke according to TOAST classification system in three Caucasians studies, no significant associations between the *TPA* -7351C/T and ischemic stroke risk were observed in all genetic models of LAA, SVO and CE, except for the recessive model (TT versus CT+CC, OR = 1.61, 95% CI = 1.00–2.59) (Figure 4) and additive model (T versus C, OR = 1.43, 95% CI = 1.15–1.79) (Figure 5) in LAA.

### Sensitivity Analysis and Publication Bias

**Figure 6:**
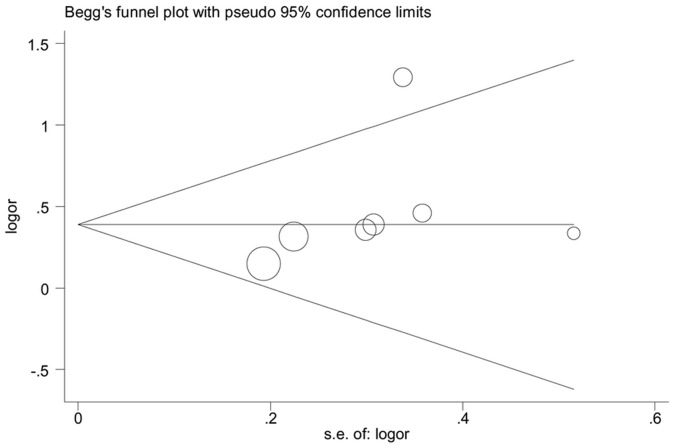
Funnel plot for publication bias in selection of studies on the TPA -7351C/T polymorphism and ischemic stroke (TT vs CT+CC).

A single study involved in the meta-analysis was deleted each time, statistically. Similar results were obtained, indicating high stability of this meta-analysis (data not shown). Publication bias was assayed by the Begg’s funnel plot and Egger’s test. The shape of the funnel plots was seemed symmetrical in recessive model (TT vs. CT+CC) (Figure 6). Then, *P* values were 0.072 in Begg’s test and 0.262 in Egger’s test, separately, also suggesting no obvious publication bias.

## Discussion

Ischemic stroke is a heterogeneous disease, which is affected by a number of genetic mutations and environmental factors. Previous data suggest that fibrinolytic pathways have been involved in stroke pathogenesis [20], [21], [22]. TPA is a serine protease synthesized and released by endothelium during thrombus formation. Its local rapid release could regulate the dissolution of arterial thrombus, which is the principle for use of thrombolytic drugs [23], [24], [25]. Therefore, slowing down the local.

TPA release rates may increase the susceptibility of ischemic stroke. Genetic factors play an important role in the variance of endothelial TPA release [26]. Ladenvall et al. found −7351 within the enhancer region of the *TPA* gene shown to be strongly correlated with endothelial TPA release rates and less than half the release rate was observed in the TT homozygous as compared with the CC homozygous [4]. Possession of a T allele was shown to reduce the binding affinity of Sp1, leading to a descent of transcriptional activity and a lower TPA release rate. Jannes et al. [6] reported an increased risk of ischemic stroke for the TT genotype, however some other studies were not consistent with the finding [7], [8], [9], [10], [11], [12]. Thus, we performed this meta-analysis to comprehensively analyze these associations.

This meta-analysis of 7 case-control studies systematically evaluated the association between -7351C/T polymorphism in the *TPA* gene and ischemic stroke risk, including 2,299 ischemic stroke cases and 1,948 controls. We found that carriers of the TT homozygote was associated with significant increased risk of ischemic stroke among East-Asians,but not in South-Asians and Caucasians. Then T allele had a 33% increased risk of ischemic stroke in East-Asians and 16% in Caucasians. It is possible that different genetic backgrounds and the environment they live in may account for these differences [27]. In addition, it was also likely that studies with small sample size may have insufficient statistical power to detect a slight effect or may have generated a fluctuated risk estimate [28]. Thus, further studies are needed to assess the effect of more interactions in different ethnicities and to validate our results.

Stroke subtype in three Caucasian studies was determined using TOAST classification system, the common system for categorization of subtypes of ischemic stroke mainly based on etiology, which affects prognosis, outcome, and management [29], [30], [31]. Genetic heterogeneity may contribute to this phenotypic diversity; some well-powered genome-wide association studies (GWAS) of ischemic stroke detected heterogeneity of risk locus effects across stroke subtypes [32], [33], especially the large artery atherosclerosis. However, the biological maps being revealed by GWAS are still largely incomplete, and not all the previous candidate genes were involved in the associations identified by GWAS, such as *TPA* gene. Thus, analyzing the relationship between *TPA* genotypes and subtypes of ischemia stroke risk is an excellent perspective to better characterize the associations between genes and ischemic stroke from the standpoint of pathogenesis. Stratified analyses performed by subtypes of ischemic stroke in Caucasians indicated that a statistically significant finding between *TPA* -7351C/T polymorphisms and ischemic stroke risk was only witnessed in LAA but not in SVO and CE. However, Jannes et al. [6] and Geng et al. [16] found this polymorphism was associated with lacunar infarction according to the OCSP classification. There are several reasons that may explain the different results between us. First, the different classification system is the most important reason. The OCSP classification relies on the initial symptoms primarily, but the TOAST is based on clinical symptoms as well as results of further investigations. Not all the patients with lacunar infarction diagnosed by OCSP classification result from small vessel disease, and it is likely to due to the striato capsular infarction without cortical signs (large-vessel disease) [34]. Second, there were too little cases and controls involved in the OCSP classification for us to perform the further analysis, which could be lack of power and reliability.

Some limitations of this meta-analysis should be considered. First, given that only seven published studies were included in the meta-analysis, publication bias could potentially occur, even though we sought to find as many publications or unpublished studies as we could by means of various searching approaches, evaluated the quality of literature strictly and used explicit methods for statistical analysis to minimize the publication bias and heterogeneity, and no statistically significant publication bias was noted in our meta-analysis. Second, the stratified analyses by subtypes of ischemic stroke were only performed in three Caucasians, owing to the insufficiency information which was impossible to obtain from some studies. Third, the number of cases and controls involved in the meta-analysis was still limited, studies with larger sample size and high quality are needed to validate our results in future. Finally, the case-control studies belong to retrospective research that is subject to methodological deficiencies.

In conclusion, this meta-analysis suggested that the *TPA* -7351C/T polymorphism was associated with increased risk of ischemic stroke, especially among East-Asians compared with Caucasians, but not in South-Asians. Further stratification for stroke subtype in Caucasians showed the association between the polymorphism and LAA, but not SVO and CE. Larger and well-designed multicentric studies are still needed to be conducted in future among different ethnicities.

## Supporting Information

Figure S1 **Prisma 2009 Checklist.**
(DOC)
